# Influence of environmental conditions on the attenuation of ricin toxin on surfaces

**DOI:** 10.1371/journal.pone.0201857

**Published:** 2018-08-08

**Authors:** Joseph P. Wood, William Richter, M. Autumn Smiley, James V. Rogers

**Affiliations:** 1 U.S. Environmental Protection Agency, Office of Research and Development, National Homeland Security Research Center, Research Triangle Park, NC, United States of America; 2 Battelle Memorial Institute, Columbus, OH, United States of America; New York State Department of Health, UNITED STATES

## Abstract

Ricin is a highly-toxic compound derived from castor plant beans. Several incidents involving contamination of residences and buildings due to ricin production or dissemination have occurred in recent years. The goal of this study was to determine whether ricin bioactivity could be attenuated in reasonable time via simple modifications of the indoor environment. Attenuation was assessed on six different materials as a function of temperature, relative humidity (RH), and contact time, using both a pure and crude preparation of the toxin. Ricin bioactivity was quantified via a cytotoxicity assay, and attenuation determined as the difference in ricin recovered from test and positive controls. The results showed that pure ricin could be attenuated successfully, while the crude ricin was generally more persistent and results more variable. We found no significant attenuation in crude ricin after two weeks at typical indoor environmental conditions, except on steel. Attenuation mostly improved with increasing temperature, but the effect of RH varied. For pure ricin, heat treatments at 40°C for 5 days or 50°C for 2–3 days achieved greater than 96% attenuation on steel. In contrast, appreciable recovery of the crude ricin preparation still occurred at 40°C after two weeks.

## Introduction

Ricin is a highly-toxic, heterodimeric protein derived from beans of the castor plant (*Ricinus communis*). Ricin exerts its toxic effect by inhibiting protein synthesis in cells [1]. Based on extrapolation from primate studies, the human lethal dose is estimated to be 1–5 mg ricin by injection or inhalation of aerosol [2]. Ricin is classified by the US Centers for Disease Control and Prevention as a Category B bio-terrorism agent [3] and is also registered as a scheduled compound in the Chemical Weapons Convention [4].

Due to its high toxicity and ease of production (e.g., over 1 million tons of castor beans are processed annually for oil manufacture), the U.S. considered the inclusion of ricin in its offensive biological weapons program [5]. Intentional dissemination of ricin may occur as an aerosol or through addition to food or water [6]. Several incidents in recent years involving the use of ricin for nefarious purposes have resulted in the contamination of structures [7]. In a more prominent example, ricin powder was processed at a Tupelo, MS, location and then sent via the US Postal Service in 2013 from Memphis, TN, to the US Capitol, the White House, and the New York City Mayor’s office. These letters had the potential to contaminate mail-sorting facilities and equipment, creating an exposure risk for those working in the area. All of these locations underwent sampling, analyses, and decontamination activities [8,9].

With respect to methods to inactivate or neutralize the ricin toxin, there has been a sizeable amount of research related to chemical treatments [10–16], as well as research for thermal treatment of foods or liquids contaminated with ricin [10,17–21]. One study demonstrated that ricin could be effectively denatured at an air temperature of 82–88°C after 24 h [22]. One commonly-cited report [23] indicates that ricin is stable for only one h at 50°C at a pH of 7.8 (implying an aqueous solution), but no data or references were provided for this assertion.

While these studies are useful and mostly provide data related to ricin associated with foods or liquids, there is minimal scientific literature describing the stability of ricin in dry form on materials or surfaces, and at typical indoor air temperatures. We found only one study that assessed the attenuation of ricin cytotoxicity (two materials) as a function of a few environmental conditions [24]. There are also limited scientific data on attenuation of ricin toxin via dry heat (air with a relative humidity [RH] < 100%) on surfaces. Nevertheless, inactivation of ricin toxin through natural attenuation processes or via mild, dry heating (up to 50°C) may be a potential option for remediation of contaminated areas. Thus, the primary objective of this study was to assess ricin toxin attenuation over time when dried on several porous and non-porous materials under multiple environmental conditions. The present study builds on a previous investigation [24], with amplification to include both crude and pure ricin preparations, additional environmental conditions, and additional, realistic materials that may be found with mail-sorting equipment or in a building. (Most biological or chemical agent persistence studies fail to account for the effect of materials or environmental matrices.[25])

Although no information is publicly available regarding the quality of the ricin preparations used in the incidents mentioned, most likely these would be of a crude quality, since producing a pure preparation would require specialized equipment and expertise. The present study includes both ricin preparations to provide a range of what could be expected to be encountered in an actual contamination scenario.

## Materials and methods

### Ricin toxin

Two preparations of ricin toxin were used in the study. Testing was conducted with a single lot of commercially prepared purified ricin toxin (Cat. No. L-1090, *Ricin communis* agglutinin II, certified as 5 mg/mL protein concentration; Vector Laboratories, Burlingame, CA), which was stored at 2–8°C and used as received. In addition, a crude preparation of the toxin was extracted from whole castor beans (Vector Laboratories, Inc.), and was prepared in the laboratory using methods derived from the scientific literature [26]. One batch of the crude form was prepared and used throughout the study. Whole castor beans were de-husked and homogenized into a slurry, precipitated from the solution, dialyzed, and rinsed with sterile PBS (Cat #D8537 Sigma-Aldrich, St. Louis, MO). The final crude ricin toxin was prepared in sterile PBS and stored at 2–8°C. While the target bio-active ricin titer for the crude preparation was also 5 mg/mL (same mass of beans used for pure and crude preparations), the actual titer averaged 6.4 mg/mL based on a cell-based bioassay and post-test statistical analyses.

### Test materials

The ricin material was deposited onto six different material types to assess the effect of material on ricin toxin stability. The porous and non-porous materials selected include those associated with mail sorting equipment (mild steel, neoprene rubber, paper, and optical grade plastic), while bare pine wood and industrial carpet were used to represent building materials. Further information on these materials is presented in Table A in S1 Supporting Information. Coupons of these materials were cut to uniform length and width (1.9 cm x 7.5 cm) from larger pieces of stock material. The coupons were sterilized prior to testing by either electron beam (E-beam) irradiation with a dose of ~200 kilograys (kGy; E-beam Services Inc., Lebanon, OH) or autoclaved at 121°C for 15 minutes. Sterilization was intended to eliminate contamination by endogenous microorganisms that might interfere with the cell-based assay used to assess ricin bioactivity.

### Environmental conditions and test matrix

Eighteen experiments were conducted overall in the study. For the initial fourteen tests, all the test materials were used. Tests were conducted at target temperatures of either 20, 25, or 30°C; and at RH levels of either 45 or 75%. Exposure periods to assess attenuation of the ricin toxin activity for Tests 1–14 ranged from 7–28 days.

In the last four experiments of the study (Tests 15–18), we assessed the attenuation of ricin activity at temperatures of 40 and 50°C, with no control of RH. (In an actual contamination incident in a building, additional heating equipment would probably be needed to reach these air temperatures, but the elevated temperatures would not be expected to be overly detrimental to the interior building materials.) In these last four tests, only mild steel was used, which allowed us to assess multiple time points in one experiment. Exposure periods for these tests ranged from 6 h to 14 days. Lastly, in Test 18, we focused only on crude ricin, as it had been attenuating less than the pure form, enabling attenuation assessment at 10 elapsed time points over a period of two weeks. Refer to Table 1 for the actual environmental test conditions for each test and Table B of S1 Supporting Information for a summary of the test matrix.

**Table 1:** Actual environmental conditions for attenuation tests.

Test	Mean ± SD Temperature °C	Mean ± SD %RH	Contact Time (Days)
1	30.09 ± 0.30	73.60 ± 2.49	7
2	25.01 ± 0.09	47.03 ± 0.30	7
3	24.99 ± 0.22	46.39 ± 1.06	14
4	25.95 ± 1.35	72.43 ± 7.20	7
5	25.58 ± 1.07	73.93 ± 5.50	14
6	29.70 ± 0.16	48.09 ± 1.73	7
7	30.03 ± 0.42	45.61 ± 3.52	14
8	30.31 ± 0.21	72.96 ± 1.16	14
9	20.41 ± 0.23	45.22 ± 1.47	7
10	20.59 ± 0.29	45.26 ± 1.40	14
11	20.80 ± 0.55	75.28 ± 1.05	7
12	20.84 ± 0.80	72.43 ± 5.14	14
13	19.80 ± 0.53	44.81 ± 4.03	21
14	19.82 ± 0.47	45.06 ± 4.18	28
15	50.26 ± 0.24	21.05 ± 2.67	0.25, 1, 1.25, 2, 3, and 4
16	39.95 ± 0.43	26.62 ± 3.31	2, 3, 4, 5, 6, 7
17	50.41 ± 0.72	19.79 ± 2.20	2, 3, 4, 5, 6, 7
18	40.37 ± 0.49	21.56 ± 2.48	3, 4, 5, 6, 7, 10, 11, 12, 13, 14

### Deposition of ricin onto material coupons

Test and positive control coupons were placed on a flat surface within a Class II biological safety cabinet (BSC; The Baker Company, Model SG603A, Sanford, MN, USA) and inoculated individually with a target mass of approximately 250 μg of either the purified or crude ricin toxin. The mass of pure ricin toxin inoculated was 250 μg per coupon based on the certified analysis of the ricin stock as received from the vendor. Actual delivered mass of crude ricin toxin per coupon material was determined using a cell-based bioassay and averaged approximately 320 μg per coupon. While this average actual quantity of crude ricin applied to each coupon was greater than our target of 250 μg, ricin attenuation was calculated based on the actual recovery from positive controls from each test. The higher than expected crude ricin toxin levels may be attributed to the variability of the cell-based assay, the variability of ricin content associated with using actual castor beans, and potential bias from additional proteins or other constituents in the crude suspension.

A 50 μL inoculum of either the purified (5 mg/mL) or crude ricin (6.4 mg/mL) stock suspension was dispensed using a micropipette and applied as a single streak across the coupon surface (Refer to Figure A in S1 Supporting Information for photograph). This technique provided decreased drying times and enabled greater distribution of the toxin across the coupon surface as compared to a single drop. After deposition, the coupons were transferred to a Class III BSC and left undisturbed to dry for approximately one h (or until visually dry) under ambient conditions, typically at 22°C and 40% RH.

The number and type of replicate coupons per material, ricin preparation, and experiment were as follows:

- Five test coupons (inoculated with ricin toxin and exposed to experimental temperature/RH for the test duration)
- Five positive controls (inoculated with ricin toxin and extracted after 1 h drying time). Note some tests were started concurrently because of same environmental condition and thus shared positive controls: Tests 2 and 3; 4 and 5; 6 and 7; 9 and 10; 11 and 12; 13 and 14; and 15–18.
- One laboratory blank (not inoculated and not exposed to experimental temperature/RH)
- One procedural blank (not inoculated and exposed to experimental temperature/RH for test duration).

Approximately 1 h post-inoculation, coupons intended for attenuation testing (including blanks) were transferred into the environmental test chamber and exposed to the environmental conditions as described below. Positive controls were then extracted and analyzed.

### Test chamber

Attenuation testing was conducted inside a 38 L stainless steel chamber. For secondary containment, the test chamber was housed inside a custom acrylic compact glove box (Plas Labs, Inc., Lansing, MI) that was hard-ducted to the facility exhaust system to maintain negative pressure. Temperature was controlled using an external water bath connected to a heat exchanger within the test chamber. RH was controlled using an external water bath connected to a Nafion tube pervaporation system (Perma Pure; Lakewood, NJ) and using fixed humidity salts (Sigma-Aldrich; St. Louis, MO). Temperature and RH in the test chamber were measured using an HMT368 temperature and humidity probe (Vaisala, Inc., Woburn, MA) and controlled with a CNI-822 controller (Omega Engineering, Stamford, CT). Data were recorded every minute during the exposure time using the controller-associated Omega Engineering iLOG software.

### Coupon extraction and ricin toxin quantification

At the end of each attenuation exposure period, coupons were individually-placed in 50 mL conical polypropylene tubes containing 10 mL of sterile PBS for extraction of ricin from the material. The vials were capped, placed on their sides and agitated on an orbital shaker for 15 min at approximately 200 rpm at room temperature (~22°C). The presence of residual bioactive toxin in the test and control coupon extracts was then determined using the cytotoxicity assay described below.

The mechanism of action by which ricin toxin exerts its toxic effect is through inhibition of protein synthesis within cells [1]. Such inhibition of protein production leads to cell death. Therefore, an *in vitro* cytotoxicity assay was used to evaluate the level of bioactive ricin toxin extracted from both attenuated and positive control material coupons. The method is based on the 3-(4,5-dimethylthiazol-2-yl)-2, 5-diphenyltetrazolium bromide (MTT) assay developed by Mossman [27]. Further details for how this assay was conducted is described elsewhere [28].

To determine the concentration of ricin toxin from each test sample, a pure ricin toxin standard (Vector Laboratories, Inc.) was prepared from the commercially-available stock solution and assayed in parallel on each test plate. The pure ricin toxin stock solution was used to prepare a seven point-standard curve of absorbance versus mass of ricin toxin protein. Refer to the S1 Supporting Information Section 3 for more information on this methodology.

Throughout the study, the inherent cytotoxicity of material coupon extracts was also assessed to determine a starting dilution that could mitigate any potential confounding cytotoxic effects observed in the ricin bioassay. To account for this potential for coupon extract-induced cytotoxicity in the ricin bioassay, the dilution factor of coupon extracts exhibiting cytotoxicity of less than 20%, when compared to negative controls (cell culture medium only), was selected as the starting dilution for all test samples.

### Attenuation calculation

The attenuation of ricin was assessed by determining the mass of bioactive toxin extracted from each test coupon subjected to the specified environmental condition, as compared to the average mass of bioactive toxin extracted from the positive control coupons.

Attenuation in terms of percent reduction for a given environmental condition, material, and ricin type, was calculated as the difference between the mean positive control mass values and the mean test mass values, divided by the mean control mass values, i.e.:

$$\frac{{\bar{Massc}}_{ij}-{\bar{Masst}}_{ij}}{{\bar{Massc}}_{ij}}*100\%=attenutation$$ (1)

where Massc_*ij*_ refers to the *j* individual mass values obtained from the positive control coupons for each material *i*. Masst_*ij*_ refers to the *j* individual mass values obtained from the corresponding test coupons for each material *i*, and the overbar designates a mean value. In this study, there were five positive controls and five corresponding test coupons (i.e., *j* = 1–5) for each coupon material *i*.

In samples where no bioactive toxin was observed in any of the five test coupon extracts after attenuation, an adjusted limit of detection (LOD) value for that material was assigned. The adjusted LOD was defined as mass of ricin toxin that corresponded to the lowest dilution factor in the standard curve.

The variance of the mean percent reduction was estimated through propagation of error using Taylor series approximation. Let *S*^2^*c_i_* be the variance of the five positive control coupons, and let *S*^2^*t_i_* be the variance of the five test coupons. Then the estimated standard error (SE) of percent reduction for each material *i* is:

$$\sqrt{\frac{\frac{{{\bar{Masst}}_{i}}^{2}}{{{\bar{Massc}}_{i}}^{2}}(\frac{{S^{2}t}_{i}}{{{\bar{Masst}}_{i}}^{2}}+\frac{{S^{2}c}_{i}}{{{\bar{Massc}}_{i}}^{2}})}{5}}*100\%.$$ (2)

where the number 5 represents the number of coupon replicates in both the control and test data sets. Each average attenuation result may be reported with an associated standard deviation, or a 95% confidence interval (CI), calculated as follows:

95%CI=Attenuation±(1.96×SE) (3)

In some cases, significant differences in attenuation for the different test conditions and toxin types were assessed depending on whether the 95% CI values for each attenuation result overlapped. However, significant effects of test variables were more robustly analyzed using the statistical procedures described below.

### Statistical analyses

The use of a biological system, i.e., a cell-based assay, to quantitate ricin toxicity may have contributed to variability in results and masked the effects of test variables. Because of this, a robust statistical analysis was conducted.

The assumption of normality for the data set was more reasonable for the log-transformed attenuation values than the untransformed values. This assessment was based on comparison of the histogram and normal probability plots that were created for the residuals from the analysis of variance (ANOVA) models, for both the log-transformed and untransformed values. Thus, all models were fitted to the log-transformed values prior to analysis, using the ratio of mass of ricin for each time point relative to the mass of ricin recovered at time zero. In this method, a smaller ratio is associated with a larger attenuation. All statistical analyses were performed using SAS (Version 9.4; Cary, NC, USA).

The ANOVA models were fitted to the log-transformed ratios for each test condition (combination of temperature, RH, and time). The models included main effects for time, material, and ricin preparation (pure or crude). The models also included all pairwise interactions and the three-way interaction. The three-way interaction was significant in all the models, and thus the effect of each factor (time, material and preparation) had to be interpreted separately at each combination of the other two factors. Pairwise comparisons were performed to test for significant differences between each combination of time, material and ricin type that differed in only one parameter. Both unadjusted and Tukey-adjusted p–values were determined, but for this manuscript, the effects of test variables are reported as significant if the Tukey-adjusted p-values were less than or equal to 0.05. Refer to the S1 Supporting Information Section 4 for additional details of statistical methodology.

## Results and discussion

The attenuation of purified and crude forms of ricin toxin deposited onto porous and nonporous material coupons was evaluated under various controlled environmental conditions and elapsed times. For the eighteen tests in this evaluation, the environmental conditions ranged from 20–50°C and 20–75% RH for durations of 6 h to 28 days. Tests 1–14 examined six different material types, while Tests 15–18 examined mild steel only, but with an increased number of timed collection points at elevated temperatures. Test 18 included crude ricin only to further increase the number of collection points evaluated. The detailed results for the mean recoveries of ricin from every material in every test, for both the positive controls and test coupons, are tabulated in the S1 Supporting Information (Sections 5 and 6). For each of the tests conducted at 20–30°C (1–14), attenuation results by material are also plotted to provide a visual representation of trends and are also shown in S1 Supporting Information (Section 7).

### Environmental test conditions

The actual environmental conditions for each test are shown in Table 1 and reported as the average value ± standard deviation (SD). Actual air temperatures were within ±1°C of target, while RH was within ±3% of the target. In Tests 15–18, temperature was controlled to either 40 or 50°C, but RH was not controlled and averaged 20–27% RH.

### Recovery of ricin from positive controls

The average percent recovery for the pure and crude ricin from the positive controls varied by material and ranged from 3 to 90% for pure ricin and 17–127% for the crude ricin. These are the study-wide averages of the percent ricin recovered one hour after inoculation at laboratory ambient conditions. (Further details on the positive control recovery results may be found in Figure D of the S1 Supporting Information.) The percent recoveries were calculated based on a 250 **μ**g pure ricin inoculum and an average 320 **μ**g inoculum of the crude form. The positive control percent recoveries were generally higher for the crude form, although statistically, the average recoveries were not significantly different for the two preparations. The wood material had the lowest average recovery at 3 and 17% for pure and crude ricin, respectively, while carpet had the highest average recovery at 90 and 127% for pure and crude, respectively. The relatively low recovery from wood is mostly likely due to its porous nature. While there are no data in the literature on ricin recovery from building materials with which to compare, the relative difficulty of recovery from porous vs. non-porous materials is consistent with the recovery from positive controls inoculated with suspensions of microorganisms [29].

### Conditions required for 99% attenuation

As an indication of the stability of the toxin, over the entire study there were only seven cases (out of over 200 test combinations of ricin type, temperature, RH, material, and contact time) in which we observed ≥ 99% reduction of ricin toxin on any of the material types tested. As seen in Table 2, elevated temperature or RH was generally associated with ≥ 99% reduction of pure or crude ricin toxin. (Statistical analyses confirm the effect of these conditions.) Specifically, there were no cases in which any form of ricin was attenuated ≥ 99% at 20°C or at 25°C and 45% RH. Moreover, ricin toxicity diminished more rapidly with higher temperatures as expected and is in agreement with other studies investigating thermal effects in aqueous food mixtures [19,20]. There was only one case in which the crude ricin preparation was attenuated ≥ 99%, while attenuation ≥ 99% occurred most often on the mild steel and paper. Lastly, as further evidence of the stability of the toxin, we note that there were no samples in the study in which ricin, as a function of cell cytotoxicity, was not detected. Although it is possible that both pure and crude ricin may have been fully attenuated under one or more of the experimental conditions presented, quantifying an absolute negative result may not be possible within the limits of the cell-based assay used.

**Table 2:** Test parameter combinations demonstrating greater than 99% reduction of ricin.

Test	Ricin Form	Temp°C	%RH	Contact Time (Days)	Material	% Reduction ± CI
4	Pure	25	75	7	Mild Steel	99.87 ± 0.11
4	Crude	25	75	7	Paper	99.54 ± 0.12
5	Pure	25	75	14	Mild Steel	99.95 ± 0.03
6	Pure	30	45	7	Carpet	99.38 ± 0.37
6	Pure	30	45	7	Paper	99.83 ± 0.24
17	Pure	50	20	6	Mild Steel	99.05 ± 0.48
17	Pure	50	20	7	Mild Steel	99.92 ± 0.02

### Effect of ricin preparation and material on attenuation

**Fig 1:**
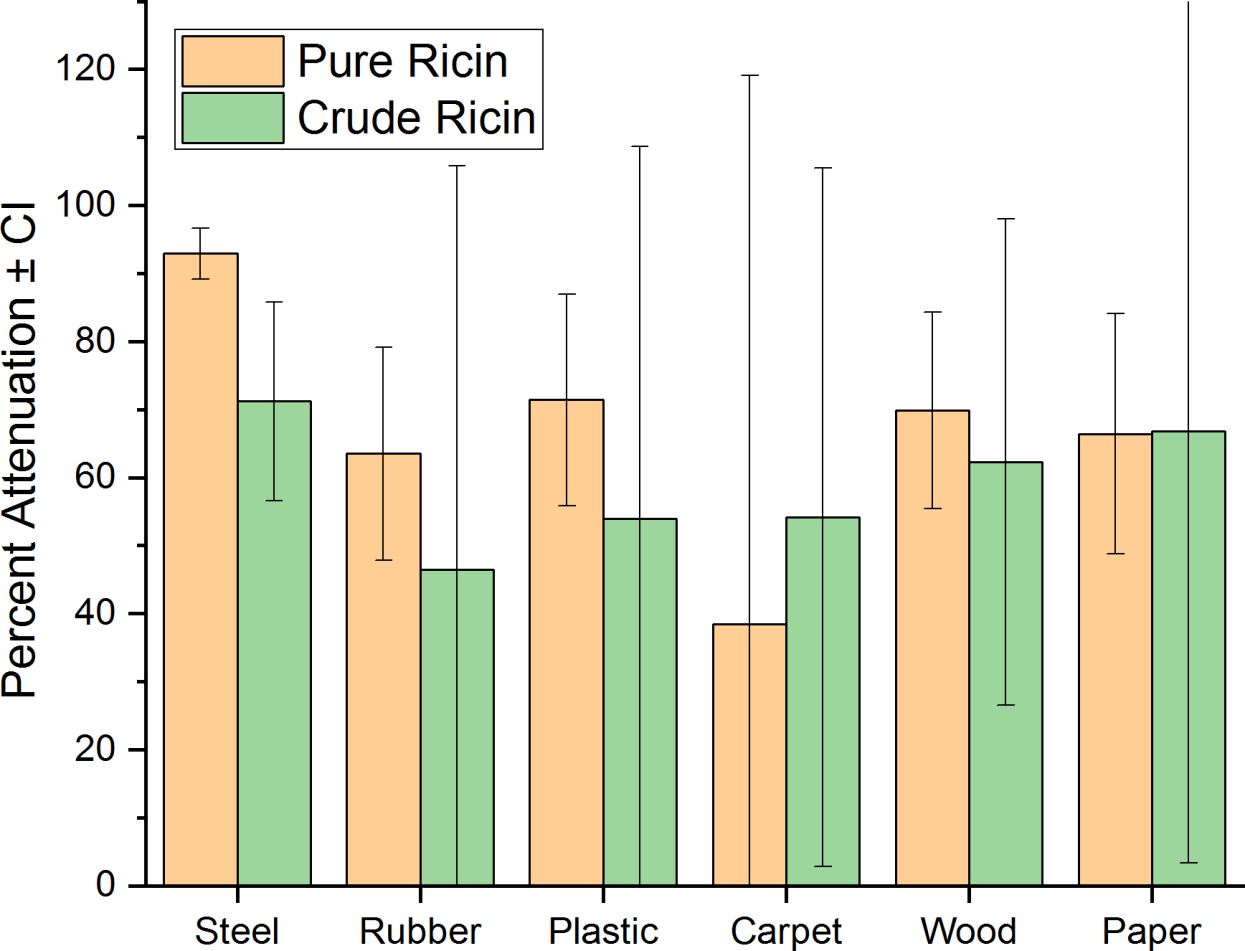
Average percent reduction for pure ricin and crude ricin by material ± standard deviation, for tests 1–14 (20–30°C).

A summary of the attenuation results, comparing the average percent reduction for the pure and crude ricin, by material, is shown in Fig 1. These results are averages for Tests 1–14, in which all materials were subject to the same environmental conditions. Overall, the average percent reductions by material ranged from 46.4 to 66.8% for crude ricin and 38.4 to 93.5% for pure ricin. The pure ricin on mild steel, neoprene rubber, optical plastic, and wood exhibited a higher average percent reduction as compared to the crude material. The average attenuation of both ricin types was highest on the steel material, although this effect was significant only for the pure form.

Similar to the positive control recovery results, these attenuation results highlight the greater variability and recovery of the crude ricin as compared to the pure ricin. This variability is most likely due to the potential for interactions of other proteins or components in solution that were not removed as they would be in the commercially-available pure material. Additionally, while the crude ricin constituents likely increased variability, they may also have shielded the ricin proteins from detrimental environmental conditions.

From the statistical analysis, the majority of pairwise comparisons (for same material, environmental condition, and elapsed time) show that there was either no significant effect of ricin type, or that the crude preparation was attenuated significantly less. There were only five cases (out of 99 comparisons) in which the crude ricin preparation was attenuated significantly more than the pure form. Interestingly, the tests at the elevated temperatures of 40 and 50°C with mild steel allowed for significant differences in the two types of ricin to become more readily apparent. With these higher temperatures, the crude form was significantly less attenuated than the pure form at all but two time points. These results indicating that the crude form is more resistant to reduction in bioactivity than a pure form of the toxin are consistent with other research in which the inactivation of ricin in vials heated to 82–88°C was investigated [22].

We postulate that the higher variability and stability of the crude ricin may be due to the presence of additional proteins and other organic materials in the crude suspension [26] that may not be found in the more refined pure form. These potentially extraneous proteins and other materials (e.g., carbohydrates, fatty acids, ash) could have mitigated the attenuating effects of the environmental conditions, as well as interfered in the quantitation assay, and are most likely absent or less prevalent in the commercially-available pure material. Additionally, although we acknowledge the use of a biological system (a cytotoxicity assay) to quantitate ricin levels may inherently produce some level of variability, the crude ricin recovery results exhibited higher variability than the pure form.

Nearly 500 pairwise comparisons between the material types were made to determine Tukey-adjusted P-values (each material was compared a total of 140 times). In most of the comparisons, there was no significant difference in attenuation between materials. However, the mild steel material did have the largest number of comparisons in which ricin was attenuated to a significantly greater extent than the other material being compared. The ranking of materials by the number of comparisons in which ricin was attenuated to a significantly greater extent than the material it was compared with (shown in parentheses) is as follows: steel (58), paper (30), carpet (24), plastic (12), rubber (7) and wood (2). From this ranking, ricin was least attenuated on wood, suggesting that the material porosity or penetrability may inhibit attenuation.

### Effect of temperature and RH on attenuation results for tests at 20–30°C (Tests 1–14)

The tests conducted at 20–30°C are representative of environmental conditions that would be expected to be achievable using the heating system of a building without any additional equipment. For each of the tests conducted at 20–30°C (1–14), attenuation results are plotted for each material and shown in Figures E-G in S1 Supporting Information. In these results one can observe the general trends of increasing attenuation over time and with increasing temperature.

In Table 3, we provide a summary of the attenuation results, by environmental condition, for the tests conducted at 20–30°C. To allow for simple comparisons, the percent reduction results were averaged across all materials for the 14-day elapsed time, since all six environmental conditions were evaluated at this time point. Fourteen days was the longest test duration investigated for the study, except for the 20°C/45% RH condition, which was tested to 28 days. The attenuation data for the 20°C/45% RH condition at 28 days are also included in Table 3 for comparison purposes.

**Table 3:** Average percent attenuation obtained for each environmental condition at 14 and 28 days.

Temperature°C	%RH	Test duration (days)	Average % Attenuation for Pure Ricin	Average % Attenuation for Crude Ricin
20	45	14	61 ± 36%	7 ± 16%
20	75	14	57 ± 32%	49 ± 37%
25	45	14	87 ± 14%	66 ± 21%
25	75	14	87 ± 15%	49 ± 52%
30	45	14	83 ± 13%	68 ± 24%
30	75	14	62 ± 32%	35 ± 42%
20	45	28	72 ± 37%	75 ± 11%

For crude ricin exposed to 20°C/45% RH, the statistical analysis showed that recovery of the toxin at 14 days was not significantly different from the positive control recoveries (i.e., there was zero to minimal attenuation; shown as 7% in Table 3) for most of the materials. Under this environmental condition (20°C/45% RH) most resembling the indoor environment, significant attenuation of the crude ricin was not observed until at least 21 days (Test 13), except for mild steel. At this same environmental condition at 4 weeks, approximately 75% of the ricin toxicity attenuated for both preparations across the range of materials. At 30°C and low RH, the pure ricin attenuated ~91% at 14 d on mild steel, which is consistent with a previous study that showed ~86% attenuation on galvanized metal for the same time [24]. We were unable to locate other similar data in the literature with which to compare our results.

From the statistical analysis (i.e., the pairwise comparisons; n = 156) to assess the effect of temperature on the attenuation of ricin, in most of the cases, there was either no significant change in attenuation (75 cases) or there was a significant increase in attenuation associated with increasing temperature (68 cases). Intuitively, the effect of increasing temperature on attenuation was more pronounced when comparing results with a 10°C temperature difference (e.g., attenuation at 20 versus 30°C).

With all other factors being equivalent, increasing the RH from 45% to 75% does not affect attenuation. In over half the pairwise comparisons, there was no significant effect on attenuation when increasing the RH. In the 33 cases where there was a significant effect of increasing the RH from 45 to 75%, 16 of those showed an increase in attenuation while 17 of the cases showed a decrease in attenuation. The effect of RH (or lack thereof) was similar for both ricin types. However, the effect of RH appears to be somewhat dependent on the material, based on the pairwise comparisons. For example, for both the mild steel and wood materials, there was either no effect of RH, or an increase in RH significantly increased the ricin attenuation in nearly all the pairwise comparisons. The opposite effect occurred with the plastic and rubber materials.

### Attenuation results for tests at elevated temperatures

**Fig 2:**
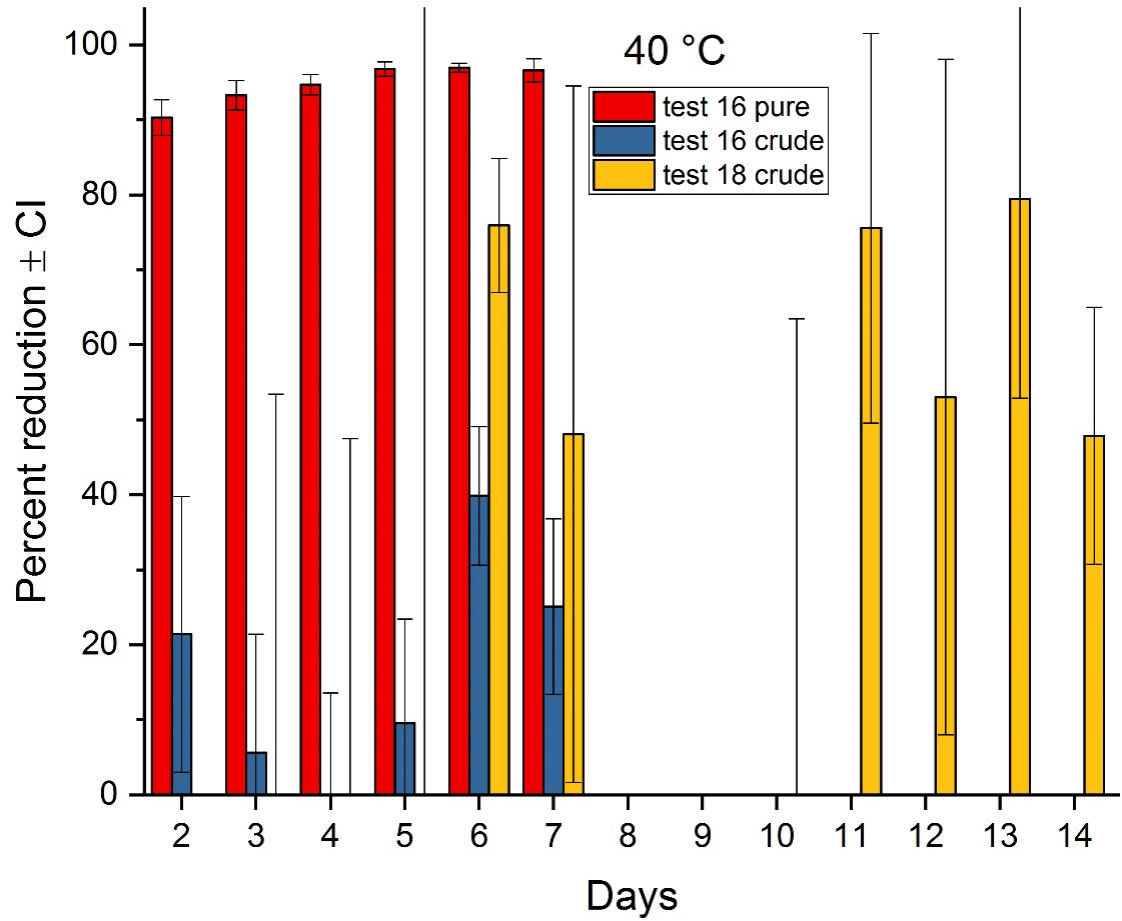
Attenuation of ricin at 40°C air temperature on mild steel as a function of time. Test 16 evaluated elapsed times from 2–7 days; Test 18 evaluated exposure times of 3–14 days.

**Fig 3:**
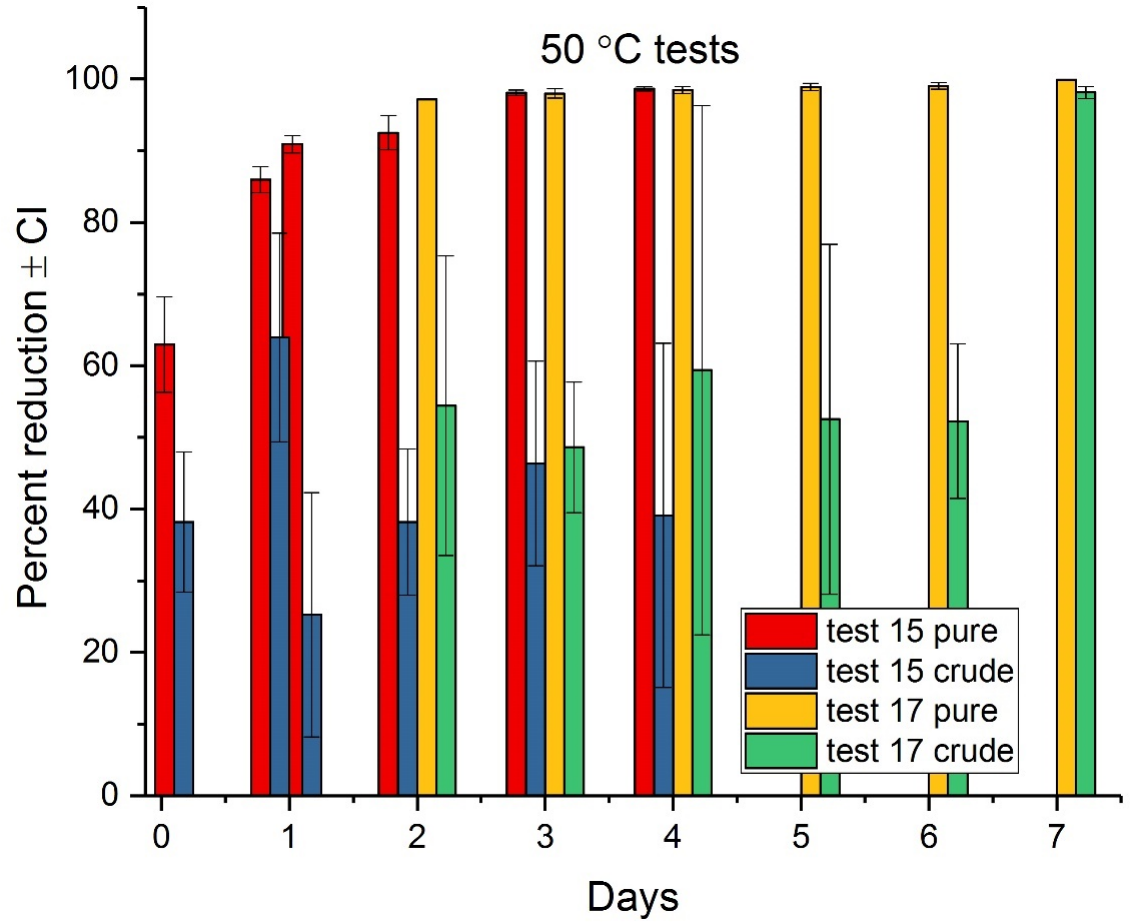
Average attenuation of ricin at 50°C on mild steel as a function of time. Test 15 evaluated elapsed times from 0.25–4.0 days; Test 17 evaluated 2–7 days.

The tests conducted at the elevated air temperatures of 40 and 50°C are representative of environmental conditions that could be achieved in a structure with additional off-the-shelf heating equipment, such as used for bed bug treatment[30]. Percent reduction results for mild steel at 40°C (Tests 16 and 18, combined) are shown in Fig 2 and results for 50°C (Tests 15 and 17, combined) are shown in Fig 3. Mild steel was selected for these tests based on its relatively lower variability in attenuation results, although this material did not diminish the variability in the crude ricin results.

The pure ricin shows steady degradation over time for both elevated temperatures, with over 90% reduction occurring within two days at both 40 and 50°C. The maximum attenuation of the pure ricin in Test 16 at 40°C was 97% and was achieved in five days, while at 50°C, the maximum reduction of 99% occurred in six days.

For the crude ricin on mild steel, attenuation over time was more subdued and variable. For example, 98% attenuation of the crude form was achieved at seven days with a temperature of 50°C; refer to Fig 3. But at the elapsed times of 0.25–6 days (both Tests 15 and 17), attenuation displayed no clear trend of increasing over time (note the variable results and overlap of the 95% CI) and ranged from approximately 25–65%. Statistically, there was no significant difference in attenuation results for the crude ricin at 50°C until day 7.

In Test 18 conducted at 40°C (Fig 2), which focused only on crude ricin on mild steel to allow for multiple test durations for up to two weeks, there was no significant attenuation of the crude ricin up to five days, while the maximum attenuation obtained was only 79%. In addition, there were five instances from the 40°C tests in which the quantity of crude ricin recovered after an elapsed time was greater than what was recovered at time zero (positive controls). This anomalous result can be attributed to the stability of the toxin, but also to the natural variability associated with the crude ricin; in these cases, the percent reduction is shown as zero in Fig 2. As with the 50°C test, this test at 40°C also demonstrates the variable crude ricin attenuation results and no significant improved attenuation for the last eight days of the test.

There are minimal data in the scientific literature with which to compare these results of attenuation of ricin via elevated air temperatures produced via dry heat. Nevertheless, one study partially resembling the present study was from Anandan et al. [10], who showed that at 50% reduction in ricin (analyzed via electrophoresis) was obtained in castor cake via dry heating at 100°C for 30 minutes.

## Supporting information

S1 Supporting Information This file contains additional information on (1) test materials; (2) the test matrix; (3) analytical method for ricin; (4) statistical analysis methods; (5) recovery of ricin from positive controls; (6) detailed attenuation results/data with average recovery of ricin from positive controls and test coupons, for every timepoint and test, and attenuation determination; and (7) figures summarizing attenuation results by material and environmental test condition.
(PDF)
