# Supplementary material for: Influence of environmental conditions on the attenuation of ricin toxin on surfaces
Source: PLoS One. 2018 Aug 8;13(8):e0201857. doi: 10.1371/journal.pone.0201857 (PMC6082540; doi:10.1371/journal.pone.0201857)
Supplement: S1 Supporting Information — This file contains additional information on (1) test materials; (2) the test matrix; (3) analytical method for ricin; (4) statistical analysis methods; (5) recovery of ricin from positive controls; (6) detailed attenuation results/data with average recovery of ricin from positive controls and test coupons, for every timepoint and test, and attenuation determination; and (7) figures summarizing attenuation results by material and environmental test condition. (PDF) [file pone.0201857.s001.pdf]

Influence of environmental conditions on the attenuation of ricin toxin on surfaces

Joseph Wood[1,*], William Richter[2], M. Autumn Smiley[2], James V. Rogers[2]

[1]U.S. Environmental Protection Agency, Office of Research and Development, National Homeland Security Research Center, Research Triangle Park, NC 27711

[2]Battelle Memorial Institute, 505 King Avenue, Columbus OH, 43201

Supporting Information

1. Test materials information

Table A. Source of materials and specifications

Material	Lot, Batch, ASTM No., or Observation	Manufacturer/ Supplier Name Location	Approximate Coupon Size, Width x Length x Thickness	Material Preparation
Mild (Carbon) Steel	Gauge 12	Adept Products, West Jefferson, OH	1.9 centimeters (cm) x 7.5 cm x 0.2 cm	Autoclave
Neoprene Rubber	Nonmarking Neoprene Rubber Part # 8837K214	McMaster Carr Aurora, OH	1.9 cm x 7.5 cm x 0.3 cm	E-Beam
Optical Grade Plastic	Optically Clear Cast Acrylic Sheet McMaster Item #8560K263	McMaster Carr Aurora, OH	1.9 cm x 7.5 cm x 0.3 cm	E-Beam
Pine Wood	Item #: 3542 Model #: 142 8PINE	Lowes Hilliard, OH	1.9 cm x 7.5 cm x 0.3 cm	E-Beam
Carpet	Shaw Swizzle EcoWorx, Style: 10401 Color: Jacks	Shaw Industries Dalton, GA	1.9 cm x 7.5 cm x 0.7 cm	E-Beam
Paper	Boise Aspen Laser Paper 24 pounds Part #BPL-2411-RC	Office MaxC Hilliard, OH	1.9 cm x 7.5 cm x 0.3 cm	E-Beam

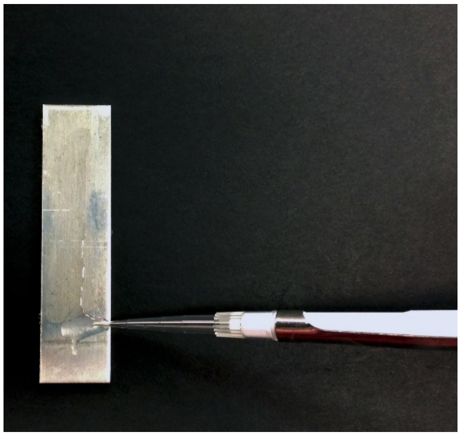

Figure A. Liquid inoculation of ricin onto coupon using a micropipette

2. Attenuation Study Test Matrix

Table B. Test matrix

Test Number	Materials	Target Temp °C	Target %RH	Time (Days)
1	Mild Steel<br>Rubber<br>Plastic<br>Wood<br>Carpet<br>Paper	30	75	7
2		25	45	7
3				14
4		25	75	7
5				14
6		30	45	7
7				14
8		30	75	14
9		20	45	7
10				14
11		20	75	7
12				14
13		20	45	21
14				28
15	Mild Steel*	50	20**	6,24,30,48,72, and 96 hours (h)
16		40		48,72,96,120,144, and 168 h
17		50		48,72,96,120,144, and 168 h
18***		40		3,4,5,6,7,10,11,12,13,14 days

Some tests were started concurrently and thus shared positive controls: Tests 2&3; 4&5; 6&7; 9&10; 11&12;13&14; and 15-18. *Only one material tested to allow for multiple time points per test.
**RH monitored but not controlled, average value shown. ***Test 18 only used crude ricin.

3. Further information on analytical method for ricin

To conduct this MTT assay, Vero cells (ATCC; Manassas, VA; kidney epithelial cells from the African green monkey) were seeded in wells of a 96-well microplate at a density of approximately 2×10^4 cells/well. Cells were then incubated for approximately 18 to 30 h at 37 ± 2 ºC under 95% air and 5% carbon dioxide and exposed to the coupon extracts (test, positive controls and blank controls) by adding 100 µL extract or test dilution to each well and performing a series of two-fold dilutions down each plate. Following 48 to 72 h exposure to sample extracts, the cells were incubated in the presence of MTT, where mitochondrial enzymes convert the yellow MTT to a purple formazan salt. The absorbance of this purple reaction product, read at 570 nanometers (nm) using a SPECTRAmax PLUS 384 microplate reader (Molecular Devices, Sunnyvale, CA), is directly proportional to the number of living cells and inversely proportional to the cytotoxic potential of ricin toxin. For all dilutions and sample transfers into the individual wells of a 96-well plate (Fisher Scientific; Pittsburgh, PA), a micropipette (Mettler-Toledo Rainin; Oakland, CA) was used, with the pipette tip replaced between wells to ensure that cross contamination did not occur.

To determine the concentration of ricin toxin from each test sample, a pure ricin toxin standard (Vector Laboratories, Inc.) was prepared from the commercially-available stock solution and assayed in parallel on each test plate. The pure ricin toxin stock solution was used to prepare a seven point-standard curve of absorbance versus calculated mass of ricin toxin protein. For each standard and test sample, absorbance values of the reference wavelength (630 nm) were subtracted from the absorbance values at 570 nm for each well. For each point used in generating the standard curve, the mean absorbance values (Y-axis) were plotted against the concentration in nanograms (ng)/mL, and a four-parameter logistic (4-PL) curve was generated by the SoftMax Pro Version 4.7 software included in the SPECTRAmax PLUS 384 microplate reader using the equation:

$$Y = \min + \frac{(\max - \min)}{1 + (X / C)^{B}} \tag{1}$$

where:

Y = absorbance %;
X = concentration of ricin ng/mL;
max = Y-value of the asymptote at the low values of X % absorbance;
min = Y-value of the asymptote at the high values of X % absorbance;
B = value related to the slope of the curve between the asymptotes;
C = X-value of the midpoint between max and min ng/mL

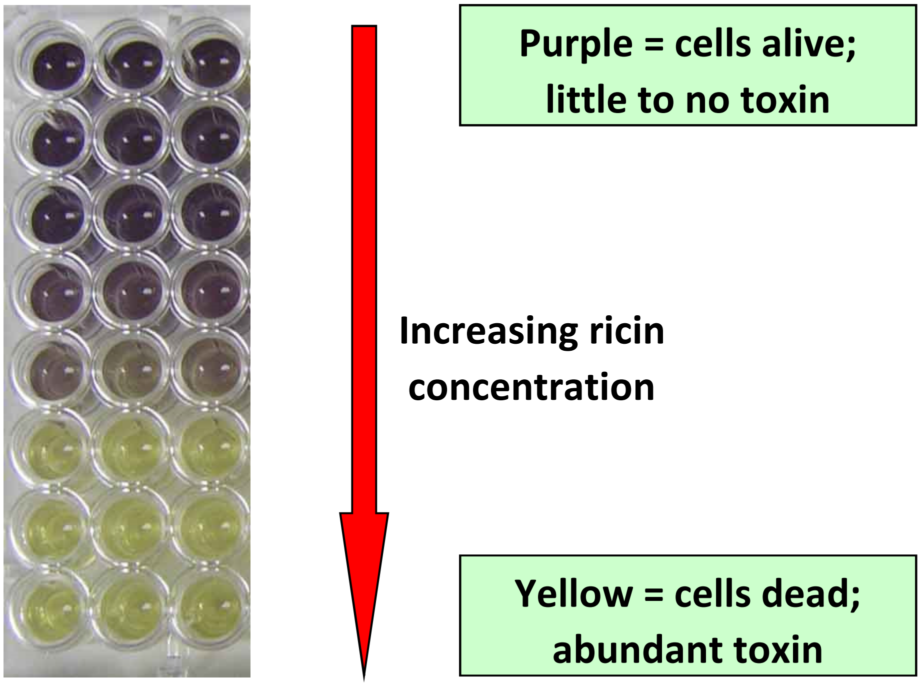

Figure B. Visual demonstration of MTT assay on a microplate

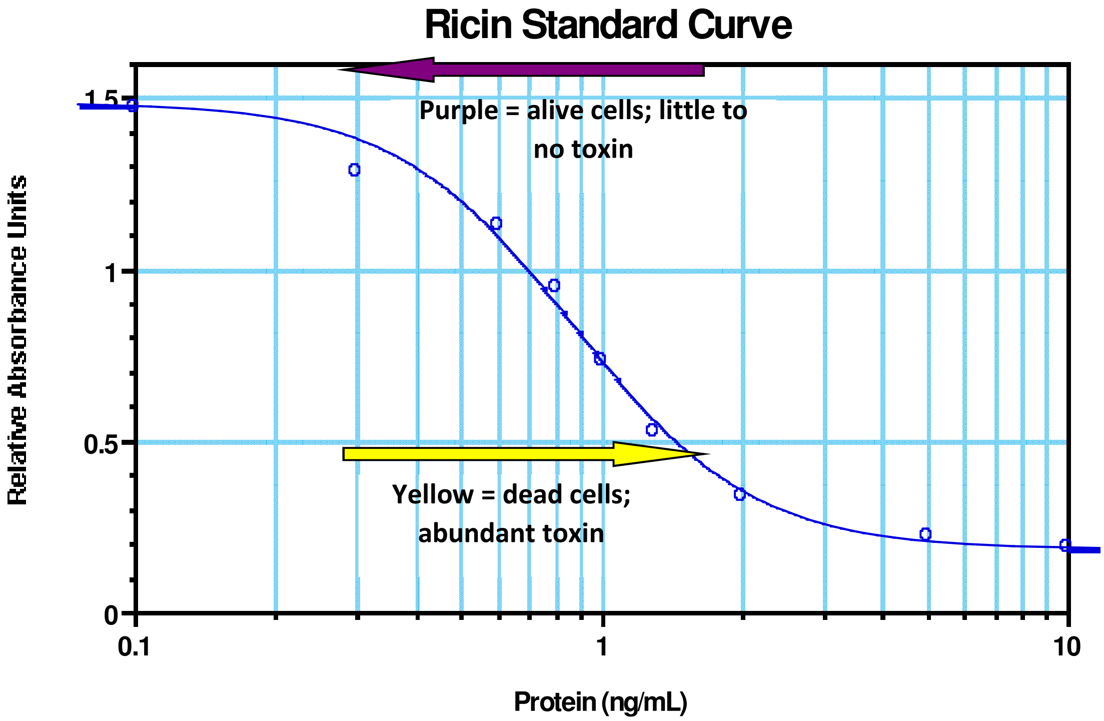

Figure C. Example of ricin cytotoxic profile with corresponding absorbance measured using a microplate reader

Throughout the study, the inherent cytotoxicity of material coupon extracts from laboratory and procedural blank coupons was assessed to determine a starting dilution that could mitigate any potential confounding cytotoxic effects observed in the ricin bioassay. To account for this potential for coupon extract-induced cytotoxicity in the ricin bioassay, the dilution factor of coupon extracts exhibiting cytotoxicity of less than 20%, when compared to negative controls (cell culture medium only), were selected as the starting dilution for all test samples. The average dilution schemes that effectively baselined the cytotoxicity of the test coupons are shown in Table S3.

Table C. Average Dilution Factors per Coupon Material

Material	Dilution Factors Required to "Zero Out" Coupon Cytotoxicity
Mild Steel	1:10
Neoprene Rubber	1:25
Optical Grade Plastic	1:8
Pine Wood	1:7
Industrial Carpet	1:135
Paper	1:8

4. Further Information on Statistical Analysis Methods

The assumption of normality for the data set was more reasonable for the log-transformed attenuation (percent reduction) values than the untransformed values. This assessment was based on comparison of the histogram and normal probability plots that were created for the residuals from the ANOVA models, for both the log-transformed and untransformed values.

Thus, all models were fitted to the log-transformed values. In addition, since the primary interest was in percent reduction from time zero, the ratio of values at each time point was taken relative to the baseline (Day 0) mean prior to analysis. The log-transformed ratio was analyzed, but results were transformed so that interpretation could be in terms of the percent reduction. A smaller ratio is associated with a larger percent reduction, and a ratio greater than one is associated with a negative percent reduction. All statistical analysis was performed using SAS (Version 9.4; Cary, NC, USA).

Analysis of variance (ANOVA) models were fitted to the log-transformed ratios for each combination of temperature and humidity. The models included main effects for time, material, and ricin preparation (pure or crude). The models also included all pairwise interactions and the three-way interaction. The following three-way ANOVA model was fitted to the base-10 log-transformed ratio response, separately for each combination of temperature and humidity:

$$Y_{ijkn} = \mu + \text{time}_i + \text{material}_j + \text{type}_k + (\text{time*material})_{ij} + (\text{time*type})_{ik} + (\text{material*type})_{jk} + (\text{time*material*type})_{ijk} + \varepsilon_{ijkn}$$

where Y_{ijkn} is the observed log-transformed, baseline-adjusted value of the nth replicate for time_i, material_j, and type_k. The parameter μ is an overall constant, time_i is the effect of time *i*, material_j is the effect of material *j*, type_k is the effect of ricin type *k*, $(\text{time*material})_{ij}$ is the interaction effect between time *i* and material *j*, $(\text{time*type})_{ik}$ is the interaction effect between time *i* and ricin type *k*, $(\text{material*type})_{jk}$ is the interaction effect between material *j* and ricin type *k*, $(\text{time*material*type})_{ijk}$ is the interaction effect between time *i*, material *j*, and ricin type *k,* and ε_{ijkn} is the random error unexplained by the model. Baseline (Day 0) results were not included in the model. The three-way interaction was significant in all of the models, and thus the effect of each factor (time, material and preparation) had to be interpreted separately at each combination of the other two factors. The models were used to estimate the percent reduction with 95 percent confidence for each combination of time, material and ricin type. In addition, pairwise comparisons were performed to test for significant differences between each combination of time, material and ricin type that differed in only one parameter. For these comparisons, unadjusted and Tukey-adjusted p–values were reported. P-values below 0.05 indicate a significant effect.

ANOVA models were also fitted separately for each combination of ricin type and material. The models included main effects for temperature, humidity, and time. The models also included all pairwise interactions and the three-way interaction. The following three-way ANOVA model was fitted to the base-10 log-transformed ratio response, separately for each combination of ricin type and material:

$$Y_{ijkn} = \mu + \text{time}_i + \text{temperature}_j + \text{humidity}_k + (\text{time*temperature})_{ij} + (\text{time*humidity})_{ik} + (\text{temperature*humidity})_{jk} + (\text{time*temperature*humidity})_{ijk} + \varepsilon_{ijkn}$$

where Y_{ijkn} is the observed log-transformed baseline-adjusted value for the nth replicate for time_i, temperature_j, and humidity_k. The parameter μ is an overall constant, time_i is the effect of time i, temperature_j is the effect of temperature j, humidity_k is the effect of humidity k, $(\text{time*temperature})_{ij}$ is the interaction effect between time i and temperature j, $(\text{time*humidity})_{ik}$ is the interaction effect between time i and humidity k, $(\text{temperature*humidity})_{jk}$ is the interaction effect between temperature j and humidity k, $(\text{time*temperature*humidity})_{ijk}$ is the interaction effect between time i, temperature j, and humidity k, and ε_{ijkn} is the random error unexplained by the model. Only results at study day 7 and study day 14 were included in the model with the exception of steel that included all available time points. The three-way interaction was significant in most of the models, and thus the effect of temperature and humidity had to be interpreted separately at each combination of the other two factors. The models were used to perform pairwise comparisons to test for significant differences between each combination of time, temperature and humidity that differed in only one parameter. For these comparisons, unadjusted and Tukey-adjusted p–values were reported. For the purposes of this report, the effects of test variables were reported as significant if the Tukey-adjusted P-values were less than or equal to 0.05.

5. Information on recovery of ricin from positive controls

The average percent recovery for the pure and crude ricin from the positive controls for each material is shown below. These are the study-wide averages of the percent ricin recovered one hour after inoculation at laboratory ambient conditions. The percent recoveries were calculated based on a 250 µg pure ricin inoculum and an average of 320 µg inoculum of the crude form.

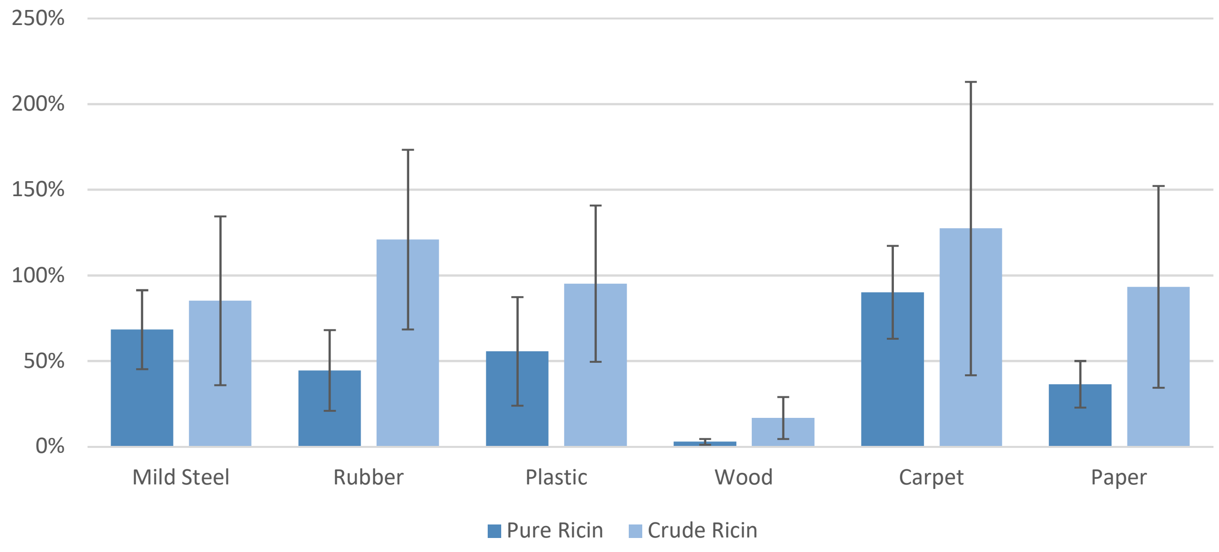

Figure D. Summary of average percent recovery from positive controls for pure ricin and crude ricin by material type, ± standard deviation. The total number of positive controls for crude and pure ricin on the steel material was 60 and 55, respectively. Forty positive controls were used overall in the study for each of the other materials, for each ricin type.

6. Detailed attenuation results with average recovery of ricin from positive controls and test materials, for every timepoint, every test; and attenuation determination.

Table D. Attenuation of Pure Ricin Toxin[a]

Test Number	Test Parameters			Material	Inoculum (µg/coupon)	Mean Recovered Ricin ± SD (µg/coupon)		%Reduction ± CI
	Temp °C±SD	%RH ±SD	Time			Positive Control	Test Coupon	
1	30.09 ± 0.30	73.60 ± 2.49	7 Days	Mild Steel	250	120.170 ± 77.058	8.140 ± 4.781	93.23 ± 5.16
				Neoprene Rubber		41.843 ± 65.194	10.764 ± 3.524	74.27 ± 35.90
				Optical Plastic		87.882 ± 79.708	3.753 ± 0.580	95.73 ± 3.44
				Bare Pine Wood		9.536 ± 1.760	3.050 ± 1.148	68.02 ± 11.75
				Industrial Carpet		241.153 ± 22.577	65.399 ±49.019	72.88 ± 17.96
				Paper		95.309 ± 34.061	5.052 ± 1.162	94.70 ± 1.97
2	25.01 ± 0.09	47.03 ± 0.30	7 Days	Mild Steel	250	180.510 ± 38.728	20.703 ± 3.678	88.53 ± 2.80
				Neoprene Rubber		56.103 ± 34.557	51.101 ± 24.325	8.92 ± 62.15
				Optical Plastic		198.226 ± 80.640	34.049 ± 3.400	82.82 ± 6.31
				Bare Pine Wood		5.377 ± 2.234	7.655 ± 2.751	[f]0.00 ± 68.55
				Industrial Carpet		154.571 ± 91.412	87.719 ± 14.440	43.25 ± 30.54
				Paper		142.943 ± 59.814	65.853 ± 39.590	53.93 ± 29.58
3	24.99 ± 0.22	46.39 ± 1.06	14 Days	Mild Steel	250	180.510 ± 38.728	6.380 ± 2.652	96.47 ± 1.45
				Neoprene Rubber		56.103 ± 34.557	10.384 ± 4.298	81.49 ± 12.04
				Optical Plastic		198.226 ± 80.640	8.358 ± 2.051	95.78 ± 1.76
				Bare Pine Wood		5.377 ± 2.234	2.081 ± 1.011	61.30 ± 21.68
				Industrial Carpet		154.571 ± 91.412	7.678 ± 3.542	95.03 ± 3.27
				Paper		142.943 ± 59.814	8.147 ± 5.807	94.30 ± 4.13
4	25.95 ± 1.35	72.43 ± 7.20	7 Days	Mild Steel	250	68.378 ± 49.618	0.089 ± 0.051	99.87 ± 0.11
				Neoprene Rubber		149.260 ± 24.963	34.536 ± 11.134	76.86 ± 7.37
				Optical Plastic		50.373 ± 60.376	38.419 ± 7.183	23.73 ± 81.10
				Bare Pine Wood		12.774 ± 9.959	4.171 ± 1.793	67.35 ± 25.48
				Industrial Carpet		206.655 ± 38.789	83.698 ± 58.006	59.50 ± 25.49
				Paper		60.340 ± 39.655	20.231 ± 4.503	66.47 ± 20.39
5	25.58 ± 1.07	73.93 ± 5.50	14 Days	Mild Steel	250	68.378 ± 49.618	0.033 ± 0.011	99.95 ± 0.03
				Neoprene Rubber		149.260 ± 24.963	11.959 ± 6.604	91.99 ± 4.05
				Optical Plastic		50.373 ± 60.376	20.927 ± 1.561	58.46 ± 43.73
				Bare Pine Wood		12.774 ± 9.959	1.759 ± 1.309	86.23 ± 13.01
				Industrial Carpet		206.655 ± 38.789	15.097 ± 10.671	92.69 ± 4.68
				Paper		60.340 ± 39.655	3.403 ± 1.829	94.36 ± 4.20

Table D. Attenuation of Pure Ricin Toxin[a] (Continued)

Test Number	Test Parameters			Material	Inoculum (µg/coupon)	Mean Recovered Ricin ± SD (µg/coupon)		%Reduction ± CI
	Temp °C	%RH	Time			Positive Control	Test Coupon	
6	29.70 ± 0.16	48.09 ± 1.73	7 Days	Mild Steel	250	159.998 ± 26.952	25.250 ± 4.137	84.22 ± 3.25
				Neoprene Rubber		167.446 ± 40.218	20.872 ± 19.731	87.54 ± 10.66
				Optical Plastic		232.860 ± 27.712	49.522 ± 4.462	78.73 ± 2.78
				Bare Pine Wood		5.396 ± 1.198	0.694 ± 0.411	87.14 ± 7.13
				Industrial Carpet		204.026 ± 98.436	1.262 ± 0.914	99.38 ± 0.47
				Paper		56.780 ± 57.697	0.094 ± 0.121	99.83 ± 0.24
7	30.03 ± 0.42	45.61 ± 3.52	14 Days	Mild Steel	250	159.998 ± 26.952	13.685 ± 5.835	91.45 ± 3.44
				Neoprene Rubber		167.446 ± 40.218	31.055 ± 6.671	81.46 ± 5.24
				Optical Plastic		232.860 ± 27.712	21.643 ± 9.633	90.71 ± 3.75
				Bare Pine Wood		5.396 ± 1.198	3.808 ± 0.775	29.44 ± 18.63
				Industrial Carpet		204.026 ± 98.436	80.311 ± 18.555	60.64 ± 18.46
				Paper		56.780 ± 57.697	4.630 ± 3.097	91.85 ± 8.69
8	30.31 ± 0.21	72.96 ± 1.16	14 Days	Mild Steel	250	90.506 ± 41.338	4.557 ± 10.096	94.97 ± 9.98
				Neoprene Rubber		56.642 ± 18.047	22.361 ± 2.860	60.52 ± 11.88
				Optical Plastic		49.017 ± 16.041	14.399 ± 3.614	70.62 ± 10.62
				Bare Pine Wood		1.938 ± 1.531	6.836 ± 2.793	[f]0.00 ± 274.96
				Industrial Carpet		147.407 ± 89.460	44.416 ± 20.943	69.87 ± 20.30
				Paper		59.617 ± 60.120	14.708 ± 2.498	75.33 ± 22.11
9	20.41 ± 0.23	45.22 ± 1.47	7 Days	Mild Steel	250	192.891 ± 37.467	48.573 ± 15.609	74.82 ± 8.29
				Neoprene Rubber		114.993 ± 36.407	101.073 ± 9.998	12.11 ± 25.55
				Optical Plastic		199.895 ± 48.331	24.371 ± 31.664	87.81 ± 14.12
				Bare Pine Wood		13.015 ± 3.962	19.645 ± 15.955	0.00 ± 114.75
				Industrial Carpet		206.481 ± 35.515	126.246 ± 69.684	38.86 ± 30.98
				Paper		138.476 ± 13.787	80.794 ± 36.946	41.65 ± 23.93
10	20.59 ± 0.29	45.26 ± 1.40	14 Days	Mild Steel	250	192.891 ± 37.467	15.611 ± 22.539	91.91 ± 10.33
				Neoprene Rubber		114.993 ± 36.407	44.091 ± 11.210	61.66 ± 13.65
				Optical Plastic		199.895 ± 48.331	43.656 ± 10.448	78.16 ± 6.51
				Bare Pine Wood		13.015 ± 3.962	31.785 ± 24.921	0.00 ± 180.04
				Industrial Carpet		206.481 ± 35.515	14.047 ± 7.134	93.20 ± 3.20
				Paper		138.476 ± 13.787	79.301 ± 24.979	42.73 ± 16.58
11	20.80 ± 0.55	75.28 ± 1.05	7 Days	Mild Steel	250	224.689 ± 96.111	5.056 ± 0.931	97.75 ± 0.92
				Neoprene Rubber		98.829 ± 19.853	86.337 ± 7.451	12.64 ± 16.74

Test Number	Temp °C	%RH	Time	Material	Inoculum (µg/coupon)	Positive Control	Test Coupon	%Reduction ± CI
				Optical Plastic		78.392 ± 8.647	89.316 ± 15.869	0.00 ± 20.89
				Bare Pine Wood		2.569 ± 0.855	11.335 ± 5.337	0.00 ± 222.93
				Industrial Carpet		291.579 ± 49.341	208.112 ± 41.463	28.63 ± 16.35
				Paper		90.726 ± 57.043	86.250 ± 9.367	4.93 ± 53.17
12	20.84 ± 0.80	72.43 ± 5.14	14 Days	Mild Steel	250	224.689 ± 96.111	5.313 ± 5.312	97.64 ± 2.25
				Neoprene Rubber		98.829 ± 19.853	34.733 ± 6.186	64.86 ± 8.27
				Optical Plastic		78.392 ± 8.647	32.356 ± 15.284	58.73 ± 17.55
				Bare Pine Wood		2.569 ± 0.855	3.913 ± 0.580	0.00 ± 48.62
				Industrial Carpet		291.579 ± 49.341	107.434 ± 30.672	63.15 ± 10.72
				Paper		90.726 ± 57.043	40.351 ± 14.248	55.52 ± 28.11
13	19.80 ± 0.53	44.81 ± 4.03	21 Days	Mild Steel	250	192.38 ± 40.482	16.393 ± 7.916	91.48 ± 3.93
				Neoprene Rubber		203.877 ± 26.250	28.306 ± 5.162	86.12 ± 2.72
				Optical Plastic		214.895 ± 33.296	27.605 ± 2.390	87.15 ± 2.00
				Bare Pine Wood		4.706 ± 1.723	1.803 ± 1.039	61.68 ± 22.93
				Industrial Carpet		349.205 ± 43.469	91.676 ± 27.681	73.75 ± 7.52
				Paper		82.853 ± 23.078	54.022 ± 20.863	47.48 ± 20.56

Table D. Attenuation of Pure Ricin Toxin[a] (Continued)

Test Number	Test Parameters			Material	Inoculum (µg/coupon)	Mean Recovered Ricin ± SD (µg/coupon)		%Reduction ± CI
	Temp °C	%RH	Time			Positive Control	Test Coupon	
14	19.82 ± 0.47	45.06 ± 4.18	28 Days	Mild Steel	250	192.38 ± 40.482	2.265 ± 1.447	98.82 ± 0.69
				Neoprene Rubber		203.877 ± 26.250	22.566 ± 6.892	88.93 ± 3.22
				Optical Plastic		214.895 ± 33.296	18.761 ± 7.392	91.27 ± 3.24
				Bare Pine Wood		4.706 ± 1.723	5.428 ± 4.933	0.00 ± 99.06
				Industrial Carpet		349.205 ± 43.469	44.936 ± 47.379	87.13 ± 11.98
				Paper		102.853 ± 23.078	34.173 ± 12.943	66.77 ± 12.82
15	50.26 ± 0.24	21.05 ± 2.67	Multiple	Mild Steel TO	250	263.835 ± 27.709	NA	NA
				Mild Steel +6 h		NA[g]	97.816 ± 17.229	62.93 ± 6.66
				Mild Steel +24 h			37.059 ± 3.819	85.95 ± 1.81
				Mild Steel +30 h			23.953 ± 2.656	90.92 ± 1.22
				Mild Steel +48 h			19.739 ± 6.912	92.52 ± 2.40
				Mild Steel +72 h			5.002 ± 0.972	98.10 ± 0.37
				Mild Steel +96 h			3.546 ± 0.834	98.66 ± 0.30
16			Multiple	Mild Steel TO	250	182.500 ± 32.073	NA	NA

				Mild Steel +48 h			17.684 ± 3.793	90.31 ± 2.36
				Mild Steel +72 h			12.289 ± 3.437	93.27 ± 1.95
	39.95 ± 0.43	26.62 ±3.31		Mild Steel +96 h		NA	9.697 ± 2.267	94.69 ± 1.36
				Mild Steel +120 h			5.913 ± 1.685	96.76 ± 0.95
				Mild Steel +144 h			5.516 ± 0.753	96.98 ± 0.59
				Mild Steel +168 h			6.180 ± 3.056	96.61 ± 1.56
				Mild Steel TO		200.912 ± 42.567	NA	NA
				Mild Steel +48 h			5.713 ± 1.310	97.16 ± .078
				Mild Steel +72 h			4.023 ± 1.167	98.00 ± 0.63
17	50.41 ± 0.72	19.79 ± 2.20	Multiple	Mild Steel +96 h	250	NA	3.102 ± 0.974	98.46 ± 0.51
				Mild Steel +120 h			2.147 ± 1.028	98.93 ± 0.49
				Mild Steel +144 h			1.902 ± 1.018	99.05 ± 0.48
				Mild Steel +168 h			0.153 ± 0.042	99.92 ± 0.02

[a] Data are expressed as the mean (± SD) of the mass of toxin recovered on five replicate individual samples, and attenuation (percent reduction ± CI).

[b] Positive Controls = samples inoculated, not attenuated.

[c] Test Coupons = samples inoculated, attenuated.

[d] CI = confidence interval (± 1.96 × standard error [SE]).

[f] As a result of high variability, negative value reported as "0".

[g] NA = Not applicable.

Table E. Attenuation of Crude Ricin Toxin[a]

Test Number	Test Parameters			Material	Inoculum (µg/coupon)	Mean Recovered Ricin ± SD (µg/coupon)		%Reduction ± CI
	Temp °C	%RH	Time			Positive Control	Test Coupon	
1	30.09 ± 0.30	73.60 ± 2.49	7 Days	Mild Steel	320	215.692 ± 83.217	3.748 ± 1.744	98.26 ± 0.92
				Neoprene Rubber		303.449 ± 135.904	213.755 ± 48.948	29.56 ± 31.06
				Optical Plastic		297.116 ± 75.901	147.589 ± 78.831	50.33 ±25.78
				Bare Pine Wood		50.730 ± 30.423	25.121 ±10.499	50.48 ± 31.73
				Industrial Carpet		331.333 ± 107.507	323.101 ± 57.961	2.48 ± 31.69
				Paper		225.850 ± 334.821	12.990 ± 2.421	94.25 ± 7.53
2	25.01 ± 0.09	47.03 ± 0.30	7 Days	Mild Steel	320	174.907 ± 29.476	104.933 ± 10.086	40.01 ± 10.20
				Neoprene Rubber		383.491 ± 25.588	221.305 ± 116.120	42.29 ± 26.76
				Optical Plastic		378.131 ± 232.700	182.085 ± 50.571	51.85 ± 28.50
				Bare Pine Wood		52.002 ± 26.692	29.364 ± 13.249	43.53 ± 33.83
				Industrial Carpet		278.805 ± 234.893	361.882 ± 65.973	[f]0.00 ± 98.07
				Paper		467.700 ± 99.403	64.740 ± 87.050	86.16 ± 16.52
3	24.99 ± 0.22	46.39 ± 1.06	14 Days	Mild Steel	320	174.907 ± 29.476	114.113 ± 42.250	34.76 ± 24.64
				Neoprene Rubber		383.491 ± 25.588	44.519 ± 8.830	88.39 ± 2.13
				Optical Plastic		378.131 ± 232.700	150.286 ± 99.070	60.26 ± 31.42
				Bare Pine Wood		52.002 ± 26.692	24.654 ± 16.321	52.59 ± 34.81
				Industrial Carpet		278.805 ± 234.893	73.737 ± 57.494	73.55 ± 26.61
				Paper		467.700 ± 99.403	57.613 ± 68.103	87.68 ± 12.97
4	25.95 ± 1.35	72.43 ± 7.20	7 Days	Mild Steel	320	13.244 ± 13.139	2.330 ± 0.890	82.41 ± 16.39
				Neoprene Rubber		217.184 ± 250.392	226.775 ± 139.189	[f]0.00 ± 119.54
				Optical Plastic		18.129 ± 11.914	5.423 ± 7.397	70.08 ± 39.70
				Bare Pine Wood		10.353 ± 4.519	12.848 ± 15.967	[f]0.00 ±143.28
				Industrial Carpet		97.020 ± 178.789	45.245 ± 95.995	53.37 ± 114.87
				Paper		335.223 ± 94.148	1.557 ± 0.068	99.54 ± 0.12
5	25.58 ± 1.07	73.93 ± 5.50	14 Days	Mild Steel	320	13.244 ± 13.139	0.511 ± 0.089	96.14 ± 3.41
				Neoprene Rubber		217.184 ± 250.392	209.061 ± 94.556	3.74 ± 104.49
				Optical Plastic		18.129 ± 11.914	66.174 ± 25.146	[f]0.00 ± 242.88
				Bare Pine Wood		10.353 ± 4.519	16.577 ± 12.620	[f]0.00 ± 123.17
				Industrial Carpet		97.020 ± 178.789	2.274 ± 0.577	97.66 ± 3.82
				Paper		335.223 ± 94.148	12.283 ± 4.807	96.34 ± 1.55

Table E. Attenuation of Crude Ricin Toxin[a] (Continued)

Test Number	Test Parameters			Material	Inoculum (µg/coupon)	Mean Recovered Ricin ± SD (µg/coupon)		%Reduction ± CI
	Temp °C	%RH	Time			Positive Control	Test Coupon	
6	29.70 ± 0.16	48.09 ± 1.73	7 Days	Mild Steel	320	258.204 ± 50.424	124.811 ± 83.041	51.66 ± 29.38
				Neoprene Rubber		510.325 ± 107.271	59.947 ± 62.351	88.25 ± 10.93
				Optical Plastic		320.984 ± 94.865	12.334 ± 14.107	96.16 ± 3.98
				Bare Pine Wood		49.605 ± 17.156	44.749 ± 31.096	9.79 ± 61.38
				Industrial Carpet		625.155 ± 137.038	172.604 ± 222.875	72.39 ± 31.70
				Paper		33.410 ± 23.788	207.509 ± 181.400	[f]0.00 ± 613.79
7	30.03 ± 0.42	45.61 ± 3.52	14 Days	Mild Steel	320	258.204 ± 50.424	43.730 ± 41.418	83.06 ± 14.36
				Neoprene Rubber		510.325 ± 107.271	188.890 ± 202.132	62.99 ± 35.38
				Optical Plastic		320.984 ± 94.865	70.771 ± 39.473	77.95 ± 12.20
				Bare Pine Wood		49.605 ± 17.156	16.510 ± 7.833	66.72 ± 17.13
				Industrial Carpet		625.155 ± 137.038	39.462 ± 19.850	93.69 ± 3.04
				Paper		33.410 ± 23.788	25.413 ± 28.179	23.94 ± 87.86
8	30.31 ± 0.21	72.96 ± 1.16	14 Days	Mild Steel	320	99.014 ± 58.918	6.000 ± 2.856	93.94 ± 4.05
				Neoprene Rubber		126.407 ± 198.446	300.259 ± 82.964	[f]0.00 ± 331.89
				Optical Plastic		193.05 ± 133.936	201.572 ± 148.937	[f]0.00 ± 92.76
				Bare Pine Wood		13.848 ± 4.799	18.291 ± 8.236	[f]0.00 ± 65.79
				Industrial Carpet		85.453 ± 35.247	49.905 ± 34.604	41.60 ± 41.30
				Paper		31.675 ± 12.263	7.440 ± 0.312	76.51 ± 8.02
9	20.41 ± 0.23	45.22 ± 1.47	7 Days	Mild Steel	320	151.614 ± 36.500	241.433 ± 47.482	[f]0.00 ± 43.39
				Neoprene Rubber		448.312 ± 162.928	481.273 ± 106.865	[f]0.00 ± 40.08
				Optical Plastic		311.765 ± 249.232	359.029 ± 23.014	[f]0.00 ± 80.95
				Bare Pine Wood		31.458 ± 8.052	88.154 ± 27.781	[f]0.00 ± 99.72
				Industrial Carpet		343.024 ± 109.283	392.103 ± 147.069	[f]0.00 ± 49.31
				Paper		337.619 ± 162.887	376.756 ± 65.259	[f]0.00 ± 50.14
10	20.59 ± 0.29	45.26 ± 1.40	14 Days	Mild Steel	320	151.614 ± 36.500	92.125 ± 29.819	39.24 ± 21.48
				Neoprene Rubber		448.312 ± 162.928	497.513 ± 147.321	[f]0.00 ± 45.60
				Optical Plastic		311.765 ± 249.232	474.946 ± 96.155	[f]0.00 ± 110.12
				Bare Pine Wood		31.458 ± 8.052	52.176 ± 18.768	[f]0.00 ± 64.18
				Industrial Carpet		343.024 ± 109.283	407.41 ± 51.057	[f]0.00 ± 35.64
				Paper		337.619 ± 162.887	333.034 ± 34.917	1.36 ± 42.69
11	20.80 ± 0.55	75.28 ± 1.05	7 Days	Mild Steel	320	507.441 ± 61.225	58.110 ± 15.498	88.55 ± 2.94
				Neoprene Rubber		651.201 ± 123.619	461.810 ± 206.330	29.08 ± 30.18

				Optical Plastic		488.828 ± 280.165	352.736 ± 53.685	27.84 ± 37.51
				Bare Pine Wood		124.416 ± 28.624	46.347 ± 10.805	62.75 ± 10.69
				Industrial Carpet		843.428 ± 221.927	585.063 ± 306.596	30.63 ± 35.65
				Paper		533.712 ± 34.554	280.187 ± 97.739	47.50 ± 16.33
12	20.84 ± 0.80	72.43 ± 5.14	14 Days	Mild Steel	320	507.441 ± 61.225	27.470 ± 8.556	94.59 ± 1.58
				Neoprene Rubber		651.201 ± 123.619	364.574 ± 79.840	44.02 ± 14.22
				Optical Plastic		488.828 ± 280.165	429.044 ± 17.369	12.23 ± 44.20
				Bare Pine Wood		124.416 ± 28.624	37.655 ± 12.467	69.73 ± 10.70
				Industrial Carpet		843.428 ± 221.927	428.759 ± 43.394	49.16 ± 12.56
				Paper		533.712 ± 34.554	327.182 ± 68.640	38.70 ± 11.80
13	19.80 ± 0.53	44.81 ± 4.03	21 Days	Mild Steel	320	327.676 ± 81.598	127.425 ± 60.295	61.11 ± 18.23
				Neoprene Rubber		453.217 ± 55.596	417.753 ± 93.573	7.82 ± 20.63
				Optical Plastic		427.380 ± 21.900	423.727 ± 46.716	0.85 ± 10.57
				Bare Pine Wood		95.760 ± 13.843	37.268 ± 11.525	61.08 ± 11.65
				Industrial Carpet		655.811 ± 167.921	36.078 ± 20.466	94.50 ± 3.00
				Paper		421.922 ± 51.930	139.322 ± 50.402	66.98 ± 11.06

Table E. Attenuation of Crude Ricin Toxin[a] (Continued)

Test Number	Test Parameters			Material	Inoculum (µg/coupon)	Mean Recovered Ricin ± SD (µg/coupon)		%Reduction ± CI
	Temp °C	%RH	Time			Positive Control	Test Coupon	
14	19.82 ± 0.47	45.06 ± 4.18	28 Days	Mild Steel	320	327.676 ± 81.598	124.88 ± 39.143	61.89 ± 13.37
				Neoprene Rubber		453.217 ± 55.596	141.939 ± 92.140	68.68 ± 18.14
				Optical Plastic		427.380 ± 21.900	36.307 ± 29.923	91.50 ± 6.15
				Bare Pine Wood		95.760 ± 13.843	27.814 ± 10.679	70.95 ± 10.45
				Industrial Carpet		655.811 ± 167.921	157.749 ± 88.907	75.95 ± 13.05
				Paper		421.922 ± 51.930	75.495 ± 35.585	82.11 ± 7.64
15	50.26 ± 0.24	21.05 ± 2.67	Multiple	Mild Steel TO	320	345.189 ± 30.327	NA	NA
				Mild Steel +6 h		NA[g]	213.405 ± 33.609	38.18 ± 9.77
				Mild Steel +24 h			124.504 ± 56.319	63.93 ± 14.57
				Mild Steel +30 h			258.024 ± 63.255	25.25 ± 17.06
				Mild Steel +48 h			213.280 ± 35.543	38.21 ± 10.20
				Mild Steel +72 h			185.231 ± 53.836	46.34 ± 14.28
				Mild Steel +96 h			210.139 ± 92.689	39.12 ± 24.00
16	39.95 ± 0.43	26.62 ±3.31	Multiple	Mild Steel TO	320	326.962 ± 35.215	NA	NA
				Mild Steel +48 h		NA	256.930 ± 62.722	21.42 ± 18.38

				Mild Steel +72 h			308.604 ± 48.759	5.61 ± 15.82
				Mild Steel +96 h			331.161 ± 36.136	0.00 ± 13.61
				Mild Steel +120 h			295.797 ± 40.980	9.53 ± 13.92
				Mild Steel +144 h			196.584 ± 27.157	39.88 ± 9.23
				Mild Steel +168 h			244.837 ± 34.795	25.12 ± 11.70
				Mild Steel TO		358.674 ± 48.186	NA	NA
				Mild Steel +48 h			163.327 ± 82.796	54.42 ± 20.93
				Mild Steel +72 h			184.327 ± 27.940	48.61 ± 9.12
17	50.41 ± 0.72	19.79 ± 2.20	Multiple	Mild Steel +96 h	320	NA	145.681 ± 149.787	59.38 ± 36.92
				Mild Steel +120 h			170.249 ± 97.208	52.53 ± 24.40
				Mild Steel +144 h			171.211 ± 37.702	52.27 ± 10.79
				Mild Steel +168 h			6.661 ± 3.393	98.14 ± 0.86
				Mild Steel T0		200.575 ± 44.939	NA	NA
				Mild Steel +3 Days			279.570 ± 105.000	0.00 ± 53.43
				Mild Steel +4 Days			216.049 ± 97.292	0.00 ± 47.49
				Mild Steel +5 Days			321.147 ± 381.795	0.00 ± 169.79
				Mild Steel +6 Days			48.251 ± 17.368	75.94 ± 8.94
18	40.37 ± 0.49	21.56 ± 2.48	Multiple	Mild Steel +7 Days	320	NA	104.119 ± 103.739	48.09 ± 46.47
				Mild Steel +10 Days			229.036 ± 135.947	0.00 ± 63.50
				Mild Steel +11 Days			49.024 ± 58.435	75.56 ± 25.98
				Mild Steel +12 Days			94.181 ± 100.927	53.04 ± 45.06
				Mild Steel +13 Days			41.227 ± 60.082	79.45 ± 26.57
				Mild Steel +14 Days			104.536 ± 31.373	47.88 ± 17.11

[a] Data are expressed as the mean (± SD) of the mass of toxin observed on five individual samples, and attenuation (percent reduction ± CI).

[b] Positive Controls = samples inoculated, not attenuated.

[c] Test Coupons = samples inoculated, attenuated.

[d] CI = confidence interval (± 1.96 × standard error [SE]).

[f] As a result of high variability, negative value reported as "0".

[g] NA = Not applicable.

7. Figures summarizing attenuation results by material and environmental test condition

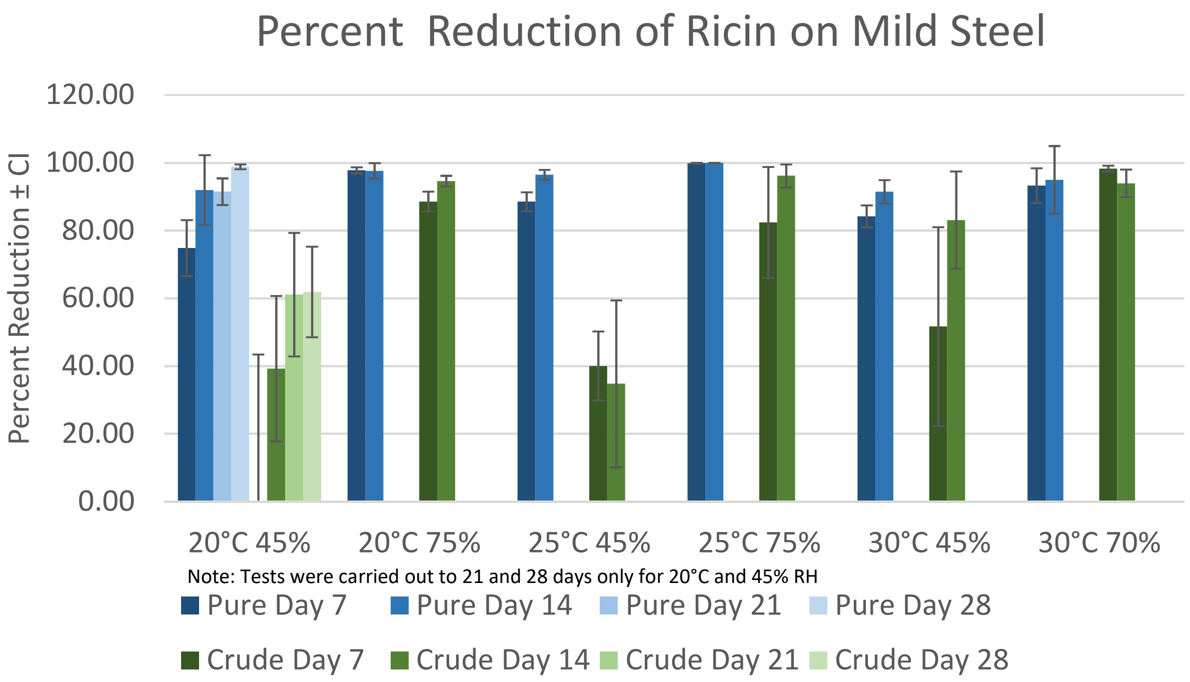

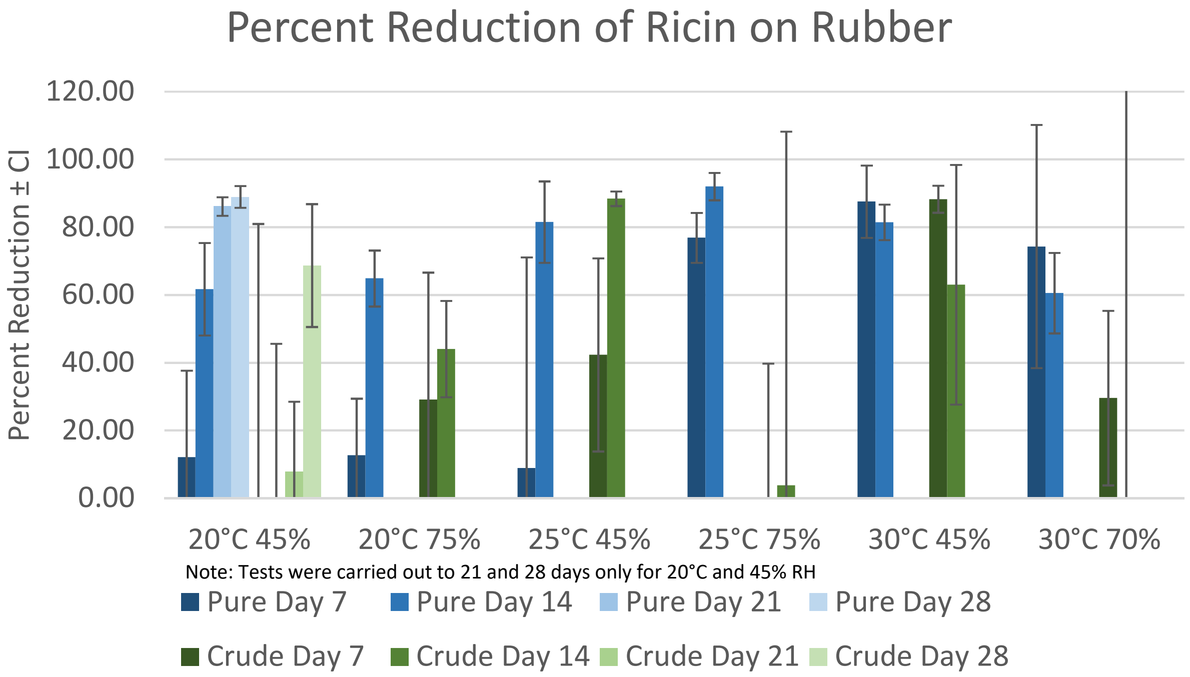

Figure E. Summary of percent reduction (Tests 1-14) results for steel and rubber, by environmental condition, comparing pure and crude ricin ± 95% confidence interval

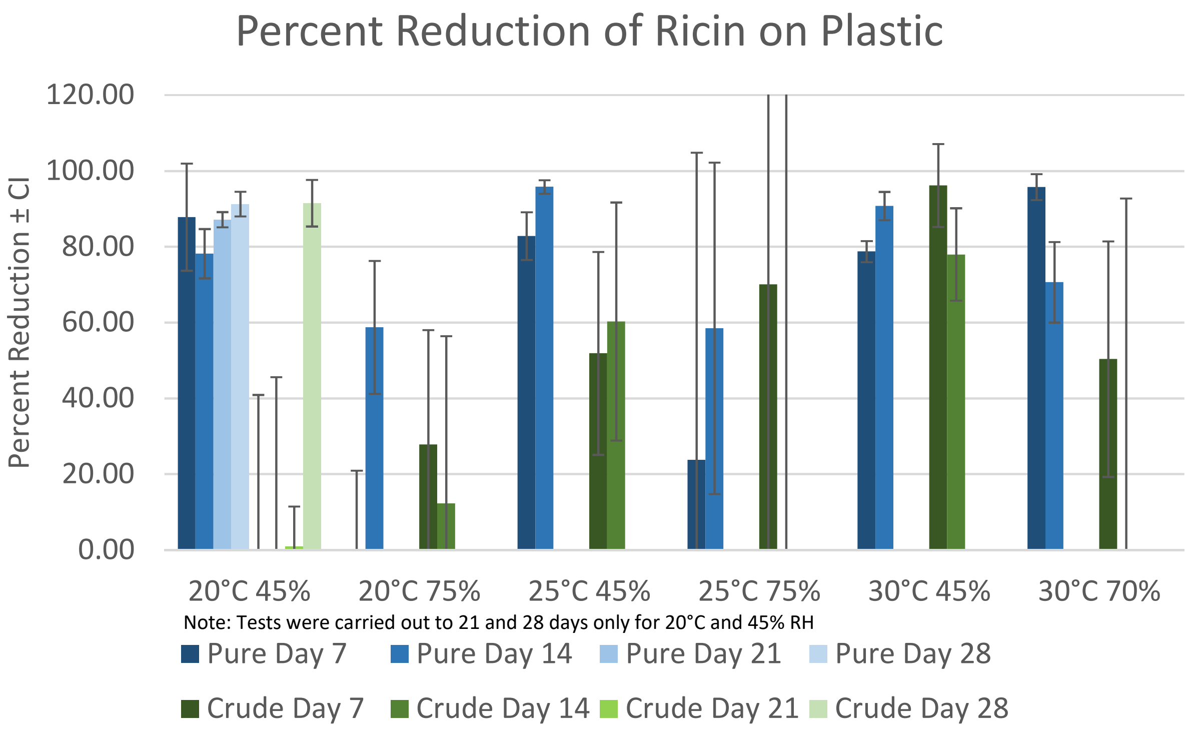

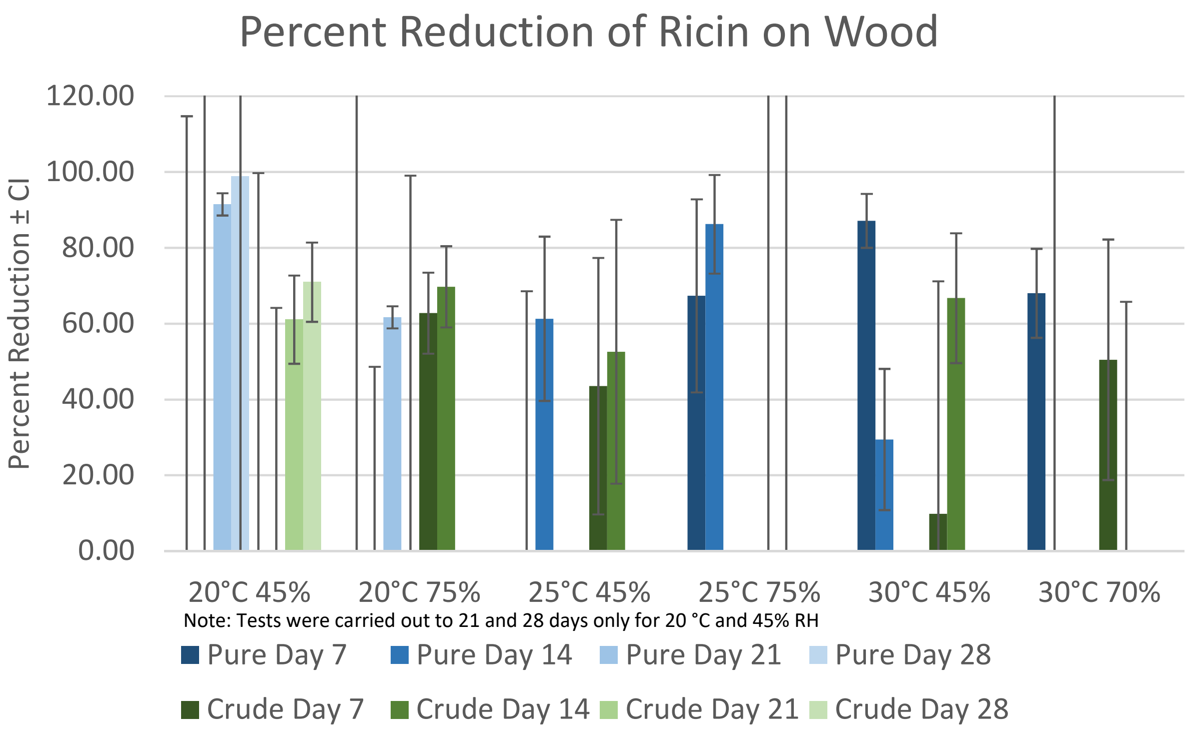

Figure F. Summary of percent reduction (Tests 1-14) results for plastic and wood, by environmental condition, comparing pure and crude ricin ± 95% confidence interval

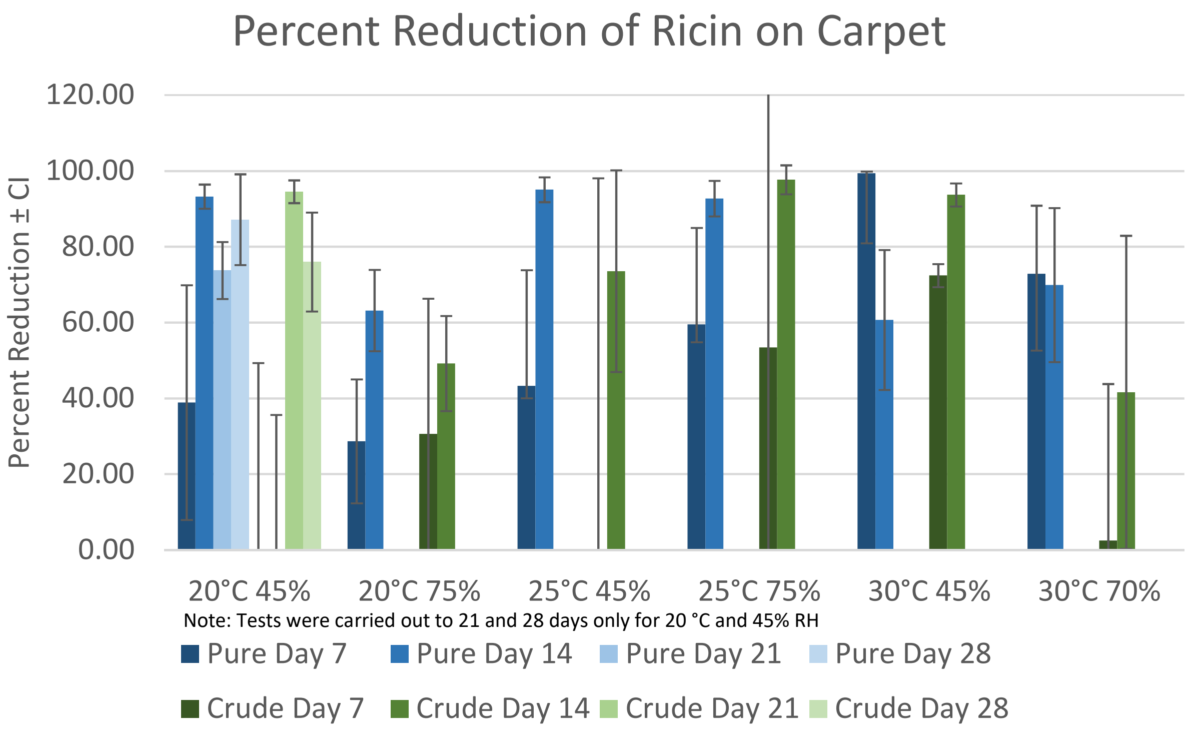

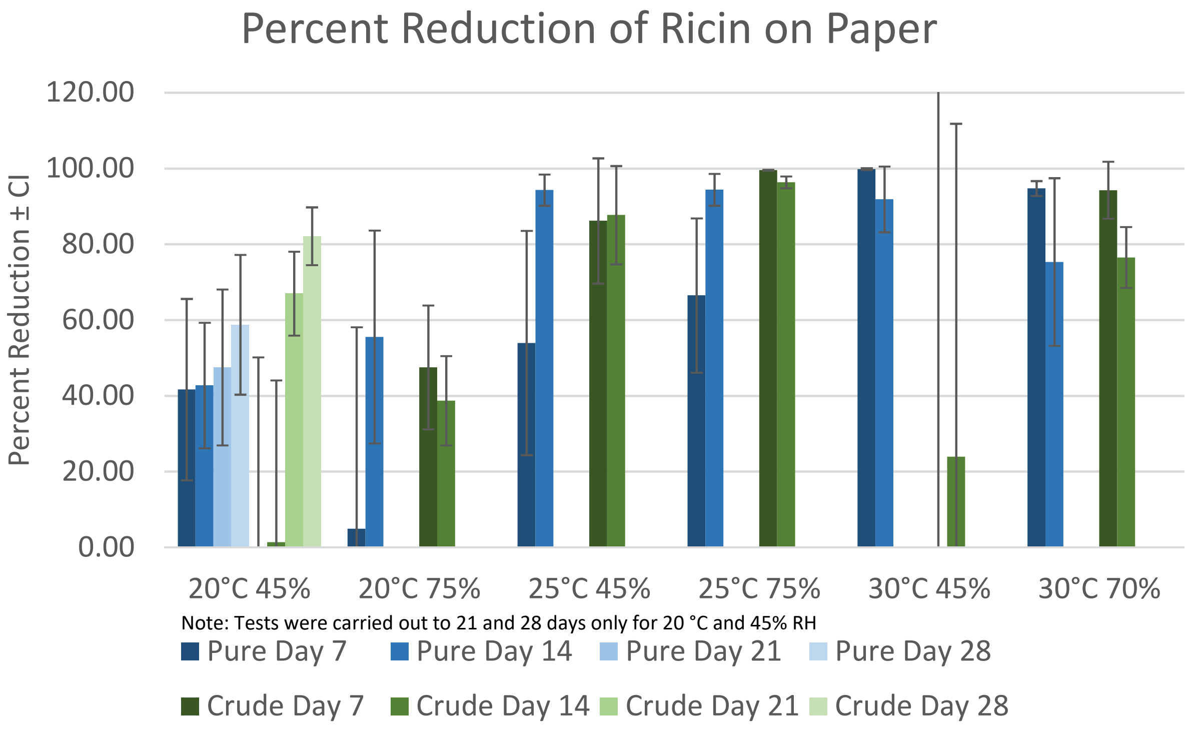

Figure G. Summary of percent reduction (Tests 1-14) results on carpet and paper, by environmental condition, comparing pure and crude ricin ± 95% confidence interval
